# Leptin and Insulin in COPD: Unveiling the Metabolic-Inflammatory Axis—A Narrative Review

**DOI:** 10.3390/jcm14082611

**Published:** 2025-04-10

**Authors:** Oana Maria Catana, Alexandra Floriana Nemes, Ramona Cioboata, Claudia Lucia Toma, Denisa Maria Mitroi, Cristina Calarasu, Costin Teodor Streba

**Affiliations:** 1 Doctoral School, University of Medicine and Pharmacy, 200349 Craiova, Romania; oana_cattana@yahoo.com (O.M.C.); denisa_maria2@yahoo.com (D.M.M.); 2 Neonatology Department, Memorial Life Hospital Bucharest, 010719 Bucharest, Romania; alexandra-floriana.nemes@drd.umfcd.ro; 3 Pneumology Department, University of Medicine and Pharmacy, 200349 Craiova, Romania; calarasu.cristina@yahoo.com (C.C.); costin.streba@umfcv.ro (C.T.S.); 4 Pneumology Department, University of Medicine Carol Davila, 020021 Bucharest, Romania

**Keywords:** chronic obstructive pulmonary disease, COPD, leptin, insulin, metabolic–inflammatory axis, oxidative stress

## Abstract

Chronic obstructive pulmonary disease (COPD) is a progressive and debilitating condition characterized by airflow limitations and systemic inflammation. The interaction between the metabolic and inflammatory pathways plays a key role in disease progression, with leptin and insulin emerging as pivotal metabolic regulators. Leptin, an adipokine that regulates energy homeostasis, and insulin, the primary regulator of glucose metabolism, are both altered in COPD patients. This narrative review provides an in-depth examination of the roles of leptin and insulin in COPD pathogenesis, focusing on the molecular mechanisms through which these metabolic regulators interact with inflammatory pathways and how their dysregulation contributes to a spectrum of extrapulmonary manifestations. These disturbances not only exacerbate COPD symptoms but also increase the risk of comorbidities such as metabolic syndrome, diabetes, cardiovascular disease, or muscle wasting. By exploring the underlying mechanisms of leptin and insulin dysregulation in COPD, this review underscores the significance of the metabolic–inflammatory axis, suggesting that restoring metabolic balance through leptin and insulin modulation could offer novel therapeutic strategies for improving clinical outcomes.

## 1. Introduction

Chronic obstructive pulmonary disease (COPD) ranks among the top three global causes of death and is responsible for 90% of fatalities in low- and middle-income countries [1]. The number of deaths from COPD increased by 17.5% between 2007 and 2017, accounting for 3.2 million in 2017 [2]. Moreover, in 2018, about 12.8 million adults (5% of the adult population) were diagnosed with COPD [3,4].

The prevalence of COPD is expected to rise due to higher smoking rates in low- and middle-income countries, aging populations in high-income nations, and increased biomass exposure, which may elevate the risk of non-smoking-related COPD and impact prevention and treatment strategies [5]. Current estimates suggest a 10.3% prevalence rate, with a 95% confidence interval (CI) of 8.2% to 12.8% [1,6]. Data modeled from the Global Burden of Disease database indicates that the number of global COPD cases in individuals aged 25 years and older is projected to increase by 23% between 2020 and 2050, potentially reaching 600 million cases by 2050 [7]. Furthermore, this increase is expected to be more pronounced among women and populations in low- and middle-income [1,8]. Additionally, research has demonstrated a connection between COPD and air pollution [9].

Therefore, the prevalence of COPD is typically higher than what health authorities estimate, making it an underdiagnosed condition. This underestimation can be attributed to a number of factors, such as a lack of reliable diagnostic criteria, variations in lung function tests, inconsistent terminology used for COPD, and a lack of government funding [10,11,12].

The recognition of significant unmet needs in the treatment of chronic obstructive pulmonary disease has led to considerable research activity in the last decade in an effort to better understand the pathophysiology behind this multifaceted disorder and enhance therapeutic options. Key concerns include the mechanisms of inflammation, metabolic dysregulation, early diagnosis, the identification of effective biomarkers, and the relationship between comorbidities and airway disease [13].

Chronic obstructive pulmonary disease is predominantly defined by airflow limitations caused by airway inflammation and remodeling, frequently linked to parenchymal destruction and the onset of emphysema [14]. Increased and progressive airway remodeling and airflow limitation contribute to a decline in exercise capacity, air trapping, and both static and dynamic hyperinflation [15]. Cigarette smoking is the leading risk factor for COPD and a significant contributor to the development of numerous chronic diseases and certain malignancies [16]. The hallmark symptom of COPD is dyspnea, and there is mounting evidence that the entirety of the symptom burden, which may also include coughing, sputum production, wheezing, and chest tightness, has a significant negative influence on quality of life and health status [17]. Currently, the international guidelines use “FEV1 (Forced expiratory volume in 1 s)/FVC (Forced vital capacity) < 70%” as the GOLD standard to diagnose COPD [18]. Although the diagnosis of COPD requires spirometric measurements, the assessment of respiratory symptoms is essential for the choice of treatment.

**Figure 1:**
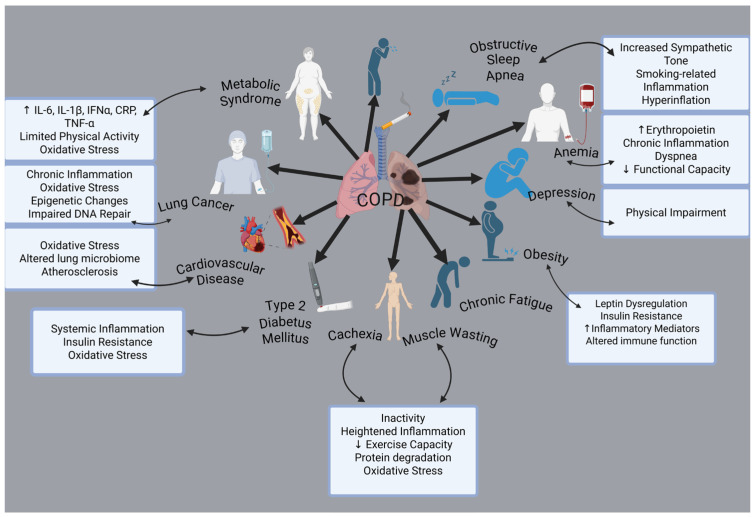
Comorbidities associated with COPD. Created with BioRender.com (accessed on 3 February 2025).

The clinical signs and pathological processes are not limited to airway remodeling and pulmonary inflammation. One of the main characteristics of COPD is that comorbidities have a big impact on an individual’s prognosis. Cardiovascular diseases, osteoporosis, muscle weakness, metabolic and endocrinological disorders, anxiety disorders, depression, and cancer are among the most prevalent health conditions associated with these factors [19] (Figure 1).

Among the primary factors potentially contributing to the elevated occurrence of co-existing conditions, systemic inflammation is widely recognized as a key feature of the disease [20]. Besides causing inflammation of the airways and lungs, smoking also enhances systemic oxidative stress, humoral and cellular inflammation, pronounced alterations in vasomotor and endothelial function, and elevated levels of circulating procoagulant factors [21]. Along with the airway and lung abnormalities commonly seen in COPD, the systemic effects of smoking may also contribute significantly to the onset of chronic diseases, either independently or in combination with other risk factors such as obesity, hyperlipidemia, and high blood pressure [22]. Additionally, individuals with stable COPD exhibit a higher prevalence of diabetes mellitus compared to the general population (15–17%) [23].

Initially, adipose tissue was thought to be an organ solely used for energy storage. However, research conducted in recent decades has shown that this conventional understanding is no longer accurate. Adipose tissue is known to play a unique role in insulin resistance due to the production of adipokines, such as leptin and adiponectin, and the rise in circulating free-fatty acids [24]. It has also been demonstrated that non-hypoxemic COPD patients exhibit higher IR (insulin resistance) than healthy individuals. Moreover, growing evidence suggests that leptin, a protein produced by adipose tissue, may play a crucial role in enhancing the inflammatory response [25]. During acute exacerbations of COPD, serum leptin levels and the leptin/adiponectin ratio are elevated, and these increases are correlated with elevated serum levels of tumor necrosis factor alpha TNF-α and IL-6 (interleukin-6) [26].

In spite of increasing evidence on the metabolic-inflammatory axis in COPD, the exact roles of leptin and insulin in disease progression remain incompletely understood. This narrative review aims to explore the interaction between leptin, insulin, and COPD, highlighting their impact on systemic inflammation, metabolic dysregulation, and potential therapeutic implications.

Additionally, this review discusses the clinical implications of these findings and emphasizes the need for personalized medicine approaches that consider metabolic dysfunction as a key component of COPD management, ultimately aiming to improve patient outcomes and quality of life. By achieving these objectives, insights into the complex relationship between the insulin–leptin axis and COPD will be provided, highlighting its potential as a therapeutic target for improving metabolic health and the well-being of patients with COPD in terms of life quality.

We conducted an extensive literature search using databases such as PubMed and Google Scholar, prioritizing peer-reviewed articles on COPD pathophysiology, metabolic dysregulation, and inflammatory pathways. As a narrative review, our primary objective is to offer a broad synthesis of emerging evidence on the insulin–leptin axis in COPD, underscoring the critical interplay between metabolic dysfunction and disease progression.

## 2. Leptin: A Metabolic and Immunological Hormone

In terms of structure, leptin is a 16 kDa non-glycosylated polypeptide that is primarily found in adipocytes, an adipokine that was first discovered to be a crucial molecule in controlling food intake and body weight [27]. In conjunction with the endocrine system and the hypothalamic–pituitary–adrenal axis, leptin plays a vital role in insulin secretion, as well as in regulating angiogenesis and energy homeostasis [28,29]. A growing amount of evidence indicates that food consumption essentially controls leptin production, and this is linked to a brief rise in ob gene (obese gene) expression, whereas fasting lowers leptin levels [30]. However, there are additional factors that can affect leptin levels; its secretion can be induced by insulin and glucocorticoids [31].

The leptin receptor (LepR) is expressed in various tissues, including adipose tissue, the heart; muscles; the lungs; the small intestine; the liver; and the central nervous system, particularly in the hypothalamus [32]. The pleiotropic effects of leptin are facilitated by the widespread expression of its receptors. Leptin is typically secreted by adipocytes into the bloodstream; it then crosses the blood–brain barrier and acts on specific areas of the brain, with particular emphasis on the hypothalamus [33]. LepR, belonging to the type I cytokine receptor family, consists of six subtypes, ranging from LepRa to LepRf [34].

Around a year after the leptin gene was discovered, it was revealed that leptin controls appetite and metabolism by inhibiting the synthesis and release of neuropeptide Y (NPY) in the arcuate nucleus (ARC) [35]. Subsequently, it was found that the dorsomedial hypothalamic nucleus (DMH), ARC, lateral hypothalamic nuclei (LH—the “hunger center”), and ventromedial hypothalamic nucleus (VMH—the “satiety center”) all contain the LEP-R isoform b (LEP-Rb), which is essential for controlling body mass and energy balance [36]. Subsequent research has shown that leptin can suppress appetite by activating neural pathways targeted by anorexigenic stimulants and inhibit those activated by orexigenic stimulants. The agouti-related protein (AgRP) and NPY are two instances of orexigenic neuropeptides. Alpha-melanocyte-stimulating hormone (α-MSH), a byproduct of proopiomelanocortin (POMC), has anorexigenic properties. The central melanocortin system, which governs energy balance, consists of neurons that express AgRP, POMC, and melanocortins [37].

The interplay between leptin signaling and the primary feeding regulation is an easy-to-understand model: leptin modulates POMC transcription, prompting the release of its α-MSH product into the synapse, where it binds to the melanocortin receptor (MCR) on neurons, thereby activating them and inhibiting appetite [38]. Leptin also prevents neurons from synthesizing NPY/AgRP, which lessens the stimulatory effect of AgRP on MCR [38]. In summary, leptin controls energy homeostasis by influencing the function of NPY/AgRP and POMC neurons in the arcuate nucleus [37].

Regardless of its impact on adiposity, brain leptin signaling controls glucose homeostasis. It can be challenging to ascertain whether leptin signaling in a particular brain structure directly regulates glucose homeostasis or whether the observed effects are secondary to changes in body weight and adiposity, given that obesity is a major contributing factor to insulin resistance. When LepR is unilaterally reactivated in the arcuate nucleus of the hypothalamus of mice that are otherwise LepR-deficient, body weight and adiposity are only slightly affected, but blood glucose and hyperinsulinemia are significantly improved [39]. According to Rossi et al., the central melanocortin system regulates glucose homeostasis [40]. There is evidence that glucose homeostasis is also regulated by leptin signaling in VMH neurons. For instance, without changing body weight, selectively deactivating SOCS3 in steroidogenic factor-1 (SF1) cells improves glucose homeostasis by increasing leptin sensitivity [41].

Crucially, leptin also plays a vital part in regulating both the innate and adaptive immune system. The structural resemblance between leptin and the long-chain helical cytokine family, which comprises IL-6, IL-11, IL-12, and oncostatin M, might be the cause [42]. A reduction in plasma leptin levels usually results in compromised immune function because its immunoregulatory roles bridge nutritional health and T helper (Th)1 immune responses [43]. The involvement in the cytokine cascade, which coordinates the host defense mechanisms and innate immune response, is strongly suggested by altered leptin production during infection and inflammation [44]—its levels decrease with chronic inflammation, in contrast to the increase seen with acute inflammatory stimulation [45]. Leptin has been demonstrated to activate the Janus kinase/signal transducer and activator of transcription (JAK/STAT) pathway, just like other IL-6 family members [46]. Additionally, the suppressor-of-cytokine signaling (SOCS)-3, which suppresses STAT signaling, is expressed in response to leptin [47]. The signaling of other cytokines in the IL-6 family is also characterized by the activation of these pathways [48]. By changing the synthesis of proinflammatory and anti-inflammatory cytokines, the physiological levels of leptin can modify the body’s reaction to an inflammatory challenge.

## 3. Insulin: Role Beyond Glucose Homeostasis

Insulin, a polypeptide hormone made up of 51 amino acids, is crucial for regulating glucose balance, promoting cell growth, and controlling metabolism. Previously, it was thought that this hormone was only produced by pancreatic β cells, but new research has revealed that some central nervous system neurons also produce it in trace amounts [49]. Food intake initiates the metabolism of glucose, leading to increased insulin production by β cells and a reduction in glucagon secretion by α cells to normalize serum glucose levels. Insulin is distributed to hepatocytes and circulates throughout the body after secretion, prompting them to store glucose as glycogen.

In addition to absorbing glucose, skeletal muscle cells, and adipocytes, other key targets of circulating insulin also contribute to lowering blood glucose levels back to baseline [50]. Insulin, through the tyrosine kinase receptor pathway, facilitates glucose uptake, skeletal muscle protein synthesis, glycogenesis, and lipogenesis, similar to other polypeptide hormones [51]. The insulin receptors (IRs) located in the plasma membrane function enzymatically to transfer phosphates from ATP to tyrosine residues on intracellular target proteins [52]. Following insulin’s binding to the α subunits, the β subunits undergo phosphorylation, thereby initiating the receptor’s catalytic activity [52]. Furthermore, several intracellular proteins that regulate insulin metabolism, cell division, and gene expression are phosphorylated by the activated receptor [53].

About 70% of glucose uptake is directed to skeletal muscle [54], while the liver uses the remaining amount of glucose in an insulin-dependent manner. A rise in plasma insulin levels activates glucose uptake and utilization in skeletal muscle, while postprandial hyperglycemia triggers the pancreas to release insulin [55]. Muscle cells primarily use fats and carbohydrates as energy sources to create ATP, which is necessary for muscle function [56]. Following glucose consumption, the concentration of plasma glucose influences the release of insulin by pancreatic β cells, resulting in hyperinsulinemia. This results in a decrease in lipid utilization and a drop in the concentration of plasma free fatty acids (FFA). By triggering multiple enzymes, insulin simultaneously promotes the uptake of glucose by skeletal muscle [57].

Adipose tissue is responsible for producing approximately 10% of the insulin required for glucose absorption [58]. Therefore, especially in obese people, the biological characteristics of adipose tissue from various sites may be important in the development and progression of metabolic disorders. There have been reports that insulin controls a number of aspects of the functional development and differentiation of adipose cells [59]. Adipose tissue is predominantly unaffected by glucose absorption; however, it significantly relies on the levels of FFA released into the bloodstream by insulin. This process is essential for the optimal functioning of vital organs, including the heart [58].

Endothelial dysfunction is one of the most frequently addressed issues related to endothelial function, with insulin playing a pivotal role in this context [60]. This condition is marked by the expression of pro-inflammatory and pro-thrombotic factors, a decline in nitric oxide (NO) bioavailability, heightened oxidative stress due to increased production of reactive oxygen species (ROS), and impaired vasoreactivity [60]. By controlling the production of NO, the two main signaling cascades that are triggered by the binding of insulin to endothelial insulin receptors and mediated by phosphatidylinositol 3-kinase (PI3K) and Ras–mitogen-activated protein kinase (MAPK) is subsequently implicated in these dysfunctional processes [61].

A complex and highly integrated network mediates insulin signaling and regulates multiple processes. The insulin receptor (IR) initiates the phosphorylation of insulin receptor substrate proteins (IRS proteins), which are vital for activating two key signaling pathways [62]. The Ras–mitogen-activated protein kinase pathway regulates gene expression and works in conjunction with the PI3K pathway to promote cell growth and differentiation. In contrast, the PI3K–AKT/protein kinase B (PKB) pathway primarily governs the metabolic functions of insulin [62].

**Figure 2:**
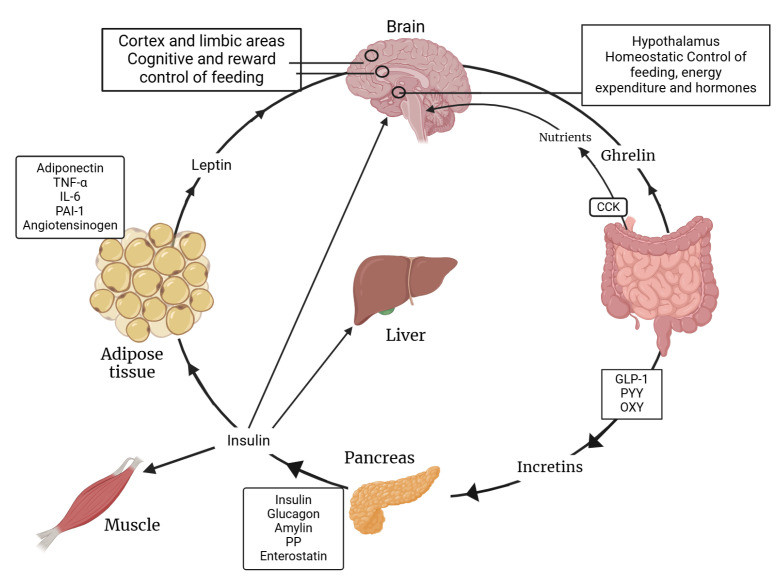
Synergy between insulin and leptin functions. PAI-1: Plasminogen Activator Inhibitor-1; PYY: Peptide YY; GLP-1: Glucagon-like Peptide-1; PP: Pancreatic Polypeptide; OXY: Oxytocin. Created with BioRender.com (accessed on 3 February 2025).

The complex interaction between insulin and leptin regarding their functions is shown below. Figure 2 illustrates how both hormones play pivotal roles in regulating energy balance and metabolism. This intricate interplay is crucial for maintaining metabolic health and understanding the mechanisms behind metabolic diseases.

## 4. Insulin–Leptin Interplay in the Central Nervous System

**Figure 3:**
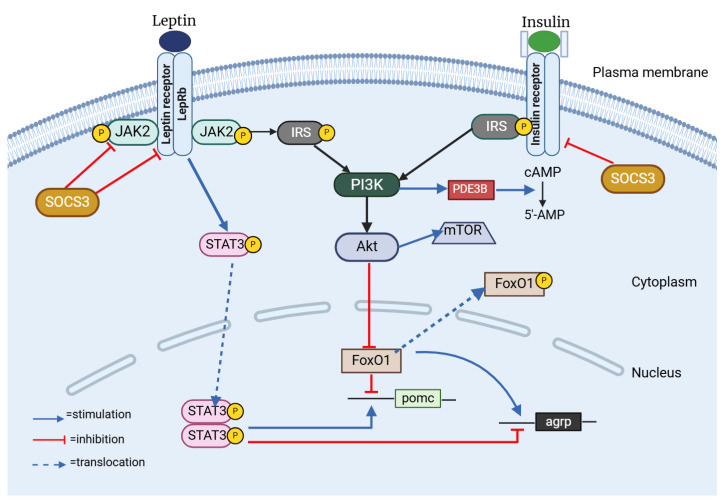
Insulin–leptin interplay in the hHypothalamus. SOCS3: Suppressor of Cytokine Signaling 3; JAK2: Janus Kinase 2; LepRb: Long isoform of Leptin Receptor B; STAT3: Signal Transducer and Activator of Transcription 3; IRS: Insulin Receptor Substrate 1; PI3K: Phosphatidylinositol 3 Kinase; FoxO1: Forkhead Box O1; mTOR: Mammalian Target of Rapamycin; PDE3B: Phosphodiesterase 3B; cAMP: cyclic Adenosine Monophosphate. Created with BioRender.com (accessed on 3 February 2025).

In the dynamic world of hypothalamic neurons, leptin and insulin are instrumental in initiating important signaling pathways. Leptin activates the JAK–STAT3 pathway, while insulin engages the PI3K–Akt pathway [63] (Figure 3). Despite mediating different but similar signaling pathways, leptin and insulin are both known to be important modulators of adiposity and energy homeostasis. Studies have indicated that obesity, insulin resistance, hyperphagia, and hyperleptinemia observed in murine models are correlated with the disruption of insulin receptors (IRs) within the central nervous system [64]. Anorexigenic effects of these hormones are mediated by CNS-expressed IRs and leptin [65,66,67]. Leptin and insulin are known to enhance the activity of the anorexigenic POMC/CART (cocaine-and amphetamine-related transcript) neurons, while simultaneously exerting an inhibitory effect on the orexigenic NPY/AgRP neurons [67,68]. Recent research has also clarified how leptin and insulin interact with one another. For instance, insulin resistance might alter leptin signaling within a hypothalamic cell line, and leptin resistance could lead to the suppression of insulin signaling [63]. Also, insulin serves a pivotal function in enhancing the activation of STAT3 mediated by leptin, which is a transcription factor of considerable importance within a crucial signaling pathway designed to inhibit the development of obesity [69].

One feature of the signaling pathways common to ObRb (Leptin Receptor B, Obese Receptor B) and IR is the engagement of PI3K, suggesting that the interaction between IRS and PI3K might serve as a mechanism linking the regulatory effects of insulin and leptin on decreasing food consumption [70,71]. Mechanistically, when insulin triggers the PI3K/Akt signaling pathway, FoxO1, its downstream mediator, is phosphorylated and it becomes inactive and moves from the nucleus to the cytoplasm, allowing STAT3 to bind to the POMC or AgRP promoter [72,73,74]. The IRS/PI3K/Akt pathway plays a critical role in the central nervous system, being fundamental to the mechanisms of insulin and leptin [65,66]. Furthermore, weight loss and an increase in leptin sensitivity are the outcomes of FoxO1 deletion in POMC neurons [75], a fact that suggests that FoxO1 may serve as a mediator in the interaction between insulin and leptin in the regulation of food intake.

Protein Tyrosine Phosphatase 1B (PTP1B) is another mediator linked to the suppression of insulin and leptin signaling. Insulin and leptin signaling are both negatively regulated by PTP1B [76,77]. By dephosphorylating the activated insulin receptor, PTP1B inhibits the activity of insulin and leptin, respectively, through JAK2 [78]. Beyond PTP1B, the suppressor of cytokine signaling 3 (SOCS3) emerges as a significant negative regulator within the intricate networks of insulin and pathways [79]. When SOCS3 is overexpressed in POMC neurons, it triggers a cascade of physiological responses that culminate in hyperphagia and the development of obesity [80,81]. Conversely, the deletion of SOCS3 in hypothalamic neurons has demonstrated improvements in leptin sensitivity, a reduction in appetite, and protection against diet-induced obesity [82]. Also, according to some theories, SOCS3 inhibits IR signaling [79].

## 5. Pathophysiological Links Between COPD, Insulin, and Leptin

### 5.1. Leptin Dysregulation in COPD

#### 5.1.1. Systemic and Local Inflammation

The mechanisms underlying lung inflammation and damage in COPD may be influenced by elevated circulating levels of leptin, given the highly vascularized nature of the lungs. A distinctive pattern of inflammation has been delineated within each compartment of the lungs. The involvement of various types of inflammatory cells, including neutrophils, dendritic cells, CD^8+^ T-lymphocytes, and macrophages, has been associated with the progression of chronic inflammation [83]. In fact, Broekhuizen et al. [84] have elucidated that leptin is identifiable in the induced sputum specimens of individuals diagnosed with mild to moderate COPD, and their findings underscore a robust correlation between the concentration of leptin in sputum and the inflammatory biomarkers TNFα and C-reactive protein (CRP).

Cigarette smoke, a major stimulus of the innate immune response, causes inflammation and propels the pathophysiology of COPD, with subsequent host defense mechanisms being modified rather than suppressed. A recent investigation has been conducted to analyze the expression of leptin within peripheral lung tissue, and it has revealed that pulmonary leptin is primarily synthesized by bronchial epithelial cells, type II pneumocytes, and alveolar macrophages [85]. Furthermore, tobacco smoke appears to serve as a potential catalyst for the expression of pulmonary leptin, as demonstrated by the significantly elevated levels of leptin-expressing bronchial epithelial cells and alveolar macrophages observed in smokers, irrespective of their COPD status, in comparison to never-smokers [85]. These results suggest the existence of a potential autocrine and/or paracrine mechanism by which leptin may modulatively influence the activation of epithelial cells in the context of COPD. In fact, numerous intracellular signal transduction pathways, such as the JAK/STAT and MAPK pathways, have been demonstrated to be activated by leptin in bronchial epithelial cells [86]. Woo et al. demonstrated that leptin causes human airway epithelial cells to produce more mucin [86], which makes it more likely that leptin contributes to mucus buildup in inflammatory lung diseases.

Compared to healthy women, women with COPD have higher serum leptin concentrations [87]. Furthermore, women with COPD have higher levels of circulating leptin than men do, and these levels rise more in proportion to an increase in body fat than in men with COPD. Breyer et al. [88] demonstrated that there is a complex relationship between low-grade systemic inflammation in COPD and adipokine metabolism, with circulating leptin, CRP, and fibrinogen having a significant relationship. The BODE index, Body mass index, degree of airflow obstruction, dyspnea, and exercise capacity (E) along with the fat-free mass index (FFMI) are clearly linked to circulating levels of leptin in COPD patients, demonstrating a positive correlation with the BODE index and a negative correlation with FFMI [89]. Additionally, in these patients, leptin appears to be the primary predictor of low FFMI [90].

#### 5.1.2. Leptin Resistance and COPD

Over one-third of individuals suffering from chronic obstructive pulmonary disease are obese [91], and obesity is linked to insulin resistance, dyslipidemia, and changes in immune function. Although the exact cause of these detrimental systemic changes in COPD is unknown, data from the COPD gene study indicate that obesity is linked to worse dyspnea, a shorter 6 min walk distance, a lower quality of life, and a higher risk of hospitalization for exacerbations [91]. Obese individuals specifically showed lower levels of Treg, which are key regulators of peripheral immune tolerance and have an inverse relationship with BMI and leptin levels [92].

Moreover, in patients with COPD who are overweight or obese, elevated insulin resistance is linked to obesity and metabolic syndrome [93]. The Bolton et al. [93] study implies that elevated levels of circulatory inflammatory mediators like IL-6 and high BMI in these patients both exacerbate insulin resistance. In individuals with COPD who present with excess body weight, there is a significant reduction in plasma adiponectin levels accompanied by markedly elevated levels of TNF-α, IL-6 and leptin [94]. Obesity in patients classified as GOLD Stages 1 and 2 is associated with a heightened risk of cardiovascular complications and overall mortality, much like the risks seen with metabolic syndrome and type 2 diabetes [95]. Conversely, overweight patients with significantly impaired lung function in GOLD Stages 3 and 4 exhibit a reduced relative mortality risk [96,97], an intriguing phenomenon commonly referred to as the “obesity paradox”.

Leptin resistance is characterized by the inability to produce its anorexigenic effects. Molecular/cellular circulatory regulation, leptin autoregulation, genetic mutations, and limited tissue access are some of the mechanisms causing this [98]. The phenomenon of central leptin resistance is a key factor in the progression of obesity, and it extends its influence to peripheral tissues, including the liver, pancreas, platelets, vasculature, and myocardium [99]. Either resistance to leptin action in specific tissues or excessive leptin activity, associated with obesity-related hyperleptinemia, may cause metabolic and inflammatory damage.

Selective leptin resistance is a subtype of leptin resistance characterized by the absence of leptin’s effects on appetite and body mass regulation, while the sympathetic nervous system remains responsive to leptin [100]. By enhancing the expression of uncoupling protein 1 (UCP1), leptin in this scenario continues to affect the sympathetic nervous system, resulting in thermogenesis in brown adipose tissue and the mobilization of lipids in white adipose tissue [101]. There are two plausible explanations: a malfunction may occur within the molecular signaling pathways responsible for mediating the effects of leptin locally rather than on a general scale, or the specific actions of leptin may be influenced by abnormalities within designated brain regions [100].

There are similarities between the mechanisms of obesity and COPD, including inflammation, and a connection between the modulation of lung function and the activation or deactivation of adipose tissue pathways. It is widely recognized that chronic obstructive pulmonary disease is linked to heightened levels of oxidative stress and reactive oxygen species, and these factors can alter signaling pathways and disrupt the function of antioxidant molecules, playing a significant role in the development of this pathology [102]. However, the association between obesity and related disorders, particularly insulin resistance and type 2 diabetes, is significantly influenced by heightened levels of reactive oxygen species [103].

### 5.2. Insulin Resistance and COPD

Insulin resistance is defined as a diminished responsiveness of insulin-targeting tissues to elevated levels of this hormone. This condition is regarded as a significant pathological contributor to a variety of health disorders, including metabolic syndrome, nonalcoholic fatty liver disease (NAFLD), atherosclerosis, and type 2 diabetes [104]. Non-physiologically elevated plasma glucose levels, the main clinical symptom of type 2 diabetes, is preceded by insulin resistance. In the prediabetic state, insulin levels increase to fulfill physiological demands, leading to chronic hyperinsulinemia and subsequent β cell dysfunction, which ultimately culminates in the development of type 2 diabetes [105].

The interaction of genetic and environmental factors contributes significantly to the pathogenesis of insulin resistance. The mechanism primarily consists of abnormalities in the immune environment, hypoxia, inflammation, lipotoxicity, and dysfunctional metabolic processes [106]. Genetic mutations affecting the insulin signaling pathway, abnormal insulin structures, substance metabolism-related genetic defects, and other related genetic defects are the categories of genetic factors linked to insulin resistance [106]. The hallmark of obesity-induced insulin resistance (IR) is compromised insulin function, which suppresses hepatic glucose production and increases muscle and adipose tissue glucose uptake [107]. It has been found that there is a causal relationship between obesity and insulin resistance and that changes in weight can either enhance or diminish insulin sensitivity [108].

IR and COPD have a pathophysiological connection, in part because they are associated with risk factors like smoking and inactivity. Furthermore, systemic effects, including inflammation and the exacerbation of physical inactivity and sedentary behavior, along with corticosteroid therapy, may also contribute to the challenges faced by patients with chronic obstructive pulmonary disease [109]. The principal mechanisms of inflammation associated with the development of insulin resistance (IR) encompass a range of inflammatory factors that markedly disrupt the signaling pathways of the insulin receptor [106]. Insulin sensitivity is directly impacted by pro-inflammatory mediators derived from macrophages, such as TNF-α and IL-1β [110]. TNF-α exerts a significant influence on insulin resistance within adipose tissue by eliciting aberrant signals on phosphorylated serine residues of insulin receptor substrate 1 (IRS1), interfering with its normal functioning, thereby compromising the integrity of insulin signaling pathways and contributing to metabolic dysfunction [111]. Furthermore, serine phosphorylation and kinase pathway defects may be two ways that TNF-α influences insulin signaling [112]. C-reactive protein is widely acknowledged as a significant indicator of inflammation that is correlated with insulin resistance (IR) and an array of metabolic disorders [106]. CRP exerts a profound influence on the regulation of energy homeostasis, insulin sensitivity, and glucose equilibrium through its interaction with leptin, thereby inhibiting the latter’s signaling pathways and altering its central actions within the hypothalamic framework [113].

According to studies (Table 1), patients with COPD had higher insulin resistance than healthy, age-matched controls [114]. A significant number of individuals diagnosed with COPD are also affected by additional chronic conditions. Inflammatory mediators present in the bloodstream can contribute to or intensify comorbidities, such as type 2 diabetes mellitus (T2DM) and metabolic syndrome [115]. This syndrome encompasses a range of cardiovascular disease risk factors, including insulin resistance [116]. Moreover, IR is not influenced by the degree of airway obstruction; therefore, even in patients with COPD who are clinically stable, it increases the risk of metabolic and cardiovascular disorders [93].

## 6. Crosstalk Between Insulin and Leptin in COPD Pathophysiology

### 6.1. Inflammation

Lung inflammation associated with COPD leads to an elevation in various biomarkers indicative of neutrophilic inflammation (neutrophils, elastase, MMP9, calprotectin, and neutrophils found in bronchoalveolar lavage) along with increased pro-inflammatory cytokines (C-reactive protein, TNF-α, IL-6, IL-1β, and IFNα within peripheral blood) [121]. The persistent increase in these inflammatory molecules leads to mild but ongoing systemic inflammation, which is significant in this scenario. In particular, cytokines such as IL-6, IL-1β, TNF-α, and CRP contribute to the development of insulin resistance [121].

Adipose tissue comprises adipocytes, macrophages, and endothelial cells, all of which have the capacity to produce and secrete various proteins: proinflammatory cytokines such as TNF-α, IL-6, and IFNγ; metabolic hormones like adiponectin, resistin, adipsin, and leptin; growth factors, including vascular endothelial growth factor (VEGF); blood pressure regulators such as PAI-1; and components of the renin–angiotensin system [121]. The pathological expansion of adipose tissue in individuals with obesity and COPD induces both adipocyte hypertrophy and hyperplasia. As adipocytes enlarge, they outstrip the available local oxygen supply and the rate of angiogenesis, and this discrepancy results in insufficient oxygenation and cellular hypoxia, subsequently triggering local inflammatory responses [122,123]. The concomitant presence of COPD and obesity has also been correlated with increased levels of IL-6, CRP, and TNF-α [121]. This correlation indicates that adipose tissue may serve as a substantial source of systemic inflammation in these individuals. Such findings illuminate the complex interactions between obesity and respiratory disorders, thereby underscoring the critical necessity for a multidimensional approach to the management of both conditions.

Cytokines released by adipocytes have a significant negative impact on metabolism and can exacerbate systemic inflammation. For instance, TNF-α manifests its effects by increasing insulin resistance through the stimulation of fatty acid release from adipose tissue into the bloodstream, directly affecting tissues such as muscle and liver [124]. Moreover, adipocytes respond to TNF-α by producing and releasing IL-6 and IL-8, which further stimulates lipolysis and the release of fatty acids from adipose tissue [125]. Also, it has been shown that TNF-α stimulates adipose tissue to produce leptin [126]. In rodent models, the expression of leptin mRNA in adipose tissue exhibits a dose-dependent increase, and the administration of endotoxins or pro-inflammatory cytokines, such as TNF-α, results in elevated concentrations of circulating leptin [127,128]. Therefore, a disrupted leptin feedback system may provide an explanation, at least partially, for elevated leptin levels, particularly during exacerbations when the systemic inflammatory response might be more pronounced than in stable patients [129]. Human airway smooth muscle cells, lung submucosa, and epithelial cells have been shown to express various leptin receptor isoforms [130]. Recent findings indicate that lung epithelial cells possess a functional leptin signaling pathway and elevated levels of leptin expression have been observed in the bronchial mucosa of patients with COPD [85].

Experimental studies suggest that airway inflammation diminishes the metabolic effects of insulin, leading to reduced glucose production in the liver and impaired glucose uptake in peripheral tissues, such as adipose tissue and muscle, ultimately affecting glucose metabolism. There were no obvious abnormalities in insulin receptor signaling that resulted in the reduced insulin action in these tissues. Accordingly, it has been shown that inflammation in the airway epithelium negatively impacts glucose metabolism by restricting blood flow to muscles and hindering microvascular recruitment without disrupting insulin signaling [131]. Moreover, elevated blood glucose levels have been correlated with the administration of corticosteroid therapy in individuals with COPD [132], and this relationship seems to be dose-dependent [133]. Since multiple studies have found no evidence of correlation between corticosteroid therapy and diabetes, there is debate regarding this topic.

### 6.2. Phenotype-Specific Pathways

COPD is a complex and heterogeneous condition characterized by multiple phenotypes, including emphysema and chronic bronchitis, each governed by distinct metabolic and inflammatory pathways influencing disease progression and comorbid burden. Recent omics-based research highlights glycerophospholipid and sphingolipid metabolism as pathways associated with worse airflow obstruction and more frequent exacerbations, whereas oxidative phosphorylation is strongly linked to the emphysematous phenotype [134]. Immune and inflammatory mechanisms also vary among phenotypes; T cell receptor signaling correlates with lung function outcomes, and antigen processing is associated with exacerbation frequency [134]. Hemostasis and immune signaling pathways, particularly in emphysema, suggest a shared immune response mechanism [135]. Additionally, gender-specific differences have been noted, with dysregulation of the phagocytosis–lysosomal axis more pronounced in female COPD patients, correlating with key clinical measures such as FEV1/FVC and disease severity [136].

Further genetic and molecular insights underscore the complexity of COPD. Microtubule transport and muscle adaptation, identified via genetic analyses, appear to be risk factors for COPD, although microtubule transport has not been previously linked to the disease [9]. Wnt signaling, a key biological pathway involved in regulating cell growth, tissue repair, and inflammation together with macrophage polarization are implicated in the development of emphysema and chronic bronchitis, reflecting their importance in inflammatory and tissue-remodeling processes [137]. Proteomic analyses point to markers such as KRT17 and DHRS9, which are highly expressed in the emphysematous phenotype and may be involved in wound healing and retinol metabolism, respectively [138]. Identifying biomarkers for specific endotypes, such as alpha-1 antitrypsin deficiency and eosinophilic COPD, could enhance targeted therapy development [138].

From a pathophysiological standpoint, the emphysema-predominant phenotype typically exhibits elevated oxidative stress, protease–antiprotease imbalance, and alveolar destruction, frequently driven by neutrophilic inflammation and high levels of reactive oxygen species. By contrast, chronic bronchitis is characterized by pronounced airway wall thickening, mucus hypersecretion, and increased levels of IL-8 and TNF-α [125]. Cachectic COPD phenotypes are marked by systemic inflammation (elevated IL-6 and TNF-α) and progressive muscle wasting, closely correlating with metabolic dysfunction and insulin resistance [121]. Meanwhile, the obese COPD phenotype shows a distinct metabolic–inflammatory pattern of elevated leptin levels, low-grade systemic inflammation, and reduced physical activity factors that collectively contribute to metabolic syndrome and elevated cardiovascular risk. These heterogeneous profiles underscore the importance of phenotype-specific therapeutic approaches targeting both the inflammatory and metabolic axes in COPD.

### 6.3. Hypoxia

In the context of COPD, there is a progressive increase in the risk of alveolar hypoxia, which subsequently leads to systemic hypoxia, as pulmonary function continues to decline over time [139]. Many of the extrapulmonary comorbidities and maladaptive processes that define COPD have been attributed to tissue hypoxia [140]. In fact, cellular metabolism and insulin sensitivity are significantly altered by prolonged hypoxia [141].

Hypoxia-inducible factor (HIF) is a transcription factor characterized by a basic helix–loop–helix structure, composed of α and β subunits, that mediates cellular responses to hypoxic conditions and has been found to promote insulin resistance in adipose tissue as a consequence of reduced oxygen availability [121]. The proline hydroxylation of HIF-α occurs in normoxic conditions, which causes the proteasome to degrade it, but hypoxia inactivates proline hydroxylases, leading to the accumulation of HIF-α and the formation of a heterodimeric transcription factor [142]. This activation subsequently reduces glucose transport in response to insulin by lowering insulin receptor phosphorylation and impairing downstream signaling mediated by Akt [143].

Hypoxia in adipose tissue results in a shift that promotes inflammation, characterized by an upregulation of adiponectin, concurrently downregulating various proinflammatory mediators, including TNF-α, IL-6, leptin, VEGF, macrophage migration inhibitory factor, tissue inhibitor of metalloproteinases-1, and monocyte chemotactic proteins [121]. Systemic hypoxia resulting from diminished pulmonary function is believed to be a key trigger for the expression of proinflammatory cytokines, alongside local adipose tissue hypoxia [144]. Chronic intermittent hypoxia is known to interfere with lipid biosynthesis [145], hinder insulin sensitivity [146], and disrupt the normal diurnal rhythm, leading to hyperglycemia and increasing the susceptibility of pancreatic β cells to damage induced by hypoxia [147].

It seems that hypoxia plays a more complex role in the liver. Despite an increase in hepatic lipid accumulation brought on by the activation of lipogenic genes [148,149], it has been demonstrated that HIF activation improves hepatic insulin sensitivity by inducing insulin receptor substrate 2 (IRS2) [150].

Insulin sensitivity is affected differently by hypoxia in skeletal muscle. Although it has been discovered that intermittent hypoxia causes insulin resistance [151], chronic hypoxia typically improves insulin action [152]. Remarkably, it has been demonstrated that skeletal muscle 5′ AMP-activated protein kinase (AMPK) pathway activation lessens the negative effects of intermittent hypoxia on whole-body glucose tolerance [152], suggesting that the adaptive response may be significantly influenced by AMPK. These results at least partially explain the positive correlation between lung function and insulin sensitivity [105].

### 6.4. Oxidative Stress

Undoubtedly, oxidative stress is a major factor in the development of COPD and may be the cause of several pathophysiological changes that occur [153]. Oxidative stress in the lungs following extended exposure to biomass fuels or cigarette smoke is most likely the primary etiologic factor driving this [154]. This stress occurs when the body’s endogenous antioxidant defenses are either genetically weakened or overwhelmed by ROS [154].

The primary source of intracellular ROS in the airway epithelial cells of patients with COPD is malfunctioning, “leaky” mitochondria [155]. Nevertheless, the xanthine/xanthine oxidase system, membrane-bound reduced nicotinamide adenine dinucleotide phosphate (NADPH) oxidases (NOX), and neutrophil-derived myeloperoxidase (MPO) are additional contributors [153]. NOX is the primary source of superoxide anions, which are comparatively weak oxidizing agents but quickly transform into more harmful ROS species like the hydroxyl radical, H_2_O_2_, or the extremely reactive peroxynitrite radicals that are produced when NO is present [156]. MPO, which is released by activated neutrophils, creates the highly toxic hypochlorous acid that combines with protein tyrosine residues to form 3-chlorotyrosine, which is found in higher concentrations in the sputum of COPD patients [157]. Intracellular antioxidant defenses protect lung cells by preventing the production of ROS and preserving redox balance. Nevertheless, a number of endogenous antioxidants are diminished in COPD, which increases oxidative stress in the lungs [158]. Through the glycoxidation of sugars and lipid peroxidation, ROS produces reactive carbonyls, which in turn form aldehydes that carbonylated proteins [159]. Smokers’ and COPD patients’ lungs have higher levels of protein carbonylation, which is correlated with the severity of the illness [160].

The abnormal redox signaling also impacts insulin metabolic signaling, endothelial dysfunction, the development of cardiovascular and renal inflammation and fibrosis, and the promotion of pro-inflammatory and pro-fibrotic pathways, all of which result in harm to the target organs [161,162]. The accumulation of oxidants, particularly in skeletal muscle and adipose tissue, is linked to the complex development of insulin resistance, with a brief surge of H_2_O_2_ generated upon insulin release, exposing cells to reactive oxygen species at low concentrations for a short duration [163]. This may augment the insulin signaling cascade through the inhibition of tyrosine phosphatase activity, thereby elevating the basal level of tyrosine phosphorylation within the insulin receptor and its subsequent targets [164]. Research has demonstrated that oxidative stress causes insulin resistance by disrupting insulin signaling [164,165]. Among the theories that have been proposed to explain insulin resistance are the accumulation of specific lipid mediators, abnormal mitochondrial activity, an increase in the stress-activated protein c-Jun-N-terminal-kinase (JNK), and inflammatory pathways [166].

Leptin, adiponectin, visfatin, resistin, apelin, and plasminogen activator inhibitor type 1 are examples of bioactive substances that regulate oxidative stress in both physiological and pathological processes [167]. In addition to increasing oxidative stress, hyperleptinemia promotes monocyte and macrophage activation and proliferation, which results in the generation of TNF-α and IL-6 [168]. TNF-α controls the immune system, inflammatory response, oxidative stress, lipid metabolism, hepatic lipogenesis, adipose cell apoptosis, and insulin signaling [169,170,171]. IL-6 is involved in the regulation of inflammation and the maintenance of energy homeostasis, and its dysregulation is associated with the progression from acute to chronic inflammatory states, particularly in the contexts of obesity and insulin resistance [172]. Additionally, leptin stimulates the synthesis of reactive intermediates like H_2_O_2_ and OH free radicals and activates NOX [173].

Oxidative stress influences many signaling pathways that initiate or promote the release of chemokines and cytokines because they contain redox-sensitive molecular targets like the transcription factor nuclear factor-kappa B (NF-kB) and different signaling molecules like protein tyrosine phosphatases, Ras/Rac, Jun N-terminal kinase, and p38 mitogen-activated protein kinase [153]. By increasing systemic pro-inflammatory cytokines and activating activated protein kinase C (PKC), the NF-κB transcription factor contributes to immune and inflammatory responses [174,175]. COPD is associated with elevated NF-kB expression and activation, which is linked to airflow limitation [176].

As COPD advances, a variety of cell types, including macrophages, neutrophils, and T cells, become hyperactivated and release proinflammatory mediators [121]. Among these mediators are TNF-α, monocyte chemotactic protein-1 (MCP-1), reactive oxygen species, as well as chemotactic factors such as leukotriene B4 (LTB4) and IL-8. This response is primarily triggered by irritants, particularly cigarette smoke [121]. The recruitment of peripheral blood monocytes, neutrophils, and CD8+ cytotoxic T cells into the airway environments is facilitated by these mediators, thereby playing a crucial role in sustaining the inflammatory response. Emphysema and tissue destruction are caused by the release of proteases by these recruited cells, especially activated neutrophils and macrophages [177,178]. The oxidative insult that results from the ROS produced by the macrophages simultaneously damages tissue and causes inflammation in the lungs. During recurrent episodes of acute exacerbation, patients diagnosed with COPD become increasingly vulnerable to bacterial and viral infections. These infections can exacerbate lung inflammation and increase the production of ROS, ultimately leading to a rapid and significant decline in lung function [179].

### 6.5. Temporal Causality, Aging, and Epigenetic Impacts

COPD is intricately linked to aging and epigenetic changes, both of which shape the question of temporal causality whether metabolic dysregulation is a precursor to COPD pathogenesis or instead a consequence of persistent inflammation and oxidative stress. Aging-related cellular senescence, driven by oxidative stress and chronic inflammation, leads to telomere attrition and cell cycle arrest, reflecting key pathways such as telomere shortening, mitochondrial dysfunction, and stem cell exhaustion [180,181]. Additionally, epigenetic age acceleration, characterized by altered deoxyribonucleic acid (DNA) methylation patterns, has been identified as a risk factor for COPD, correlating with lung function decline and disease severity [182]. Differential DNA methylation in COPD can affect gene expression and contribute to pathogenesis, while microRNAs such as miR-125a-5p influence lung epithelial cell senescence [183,184]. Epigenetic biomarkers including DNA methylation signatures and histone modifications continue to show promise for improving diagnosis and treatment strategies [180,181]. Furthermore, gene–environment interactions play a vital role in disease trajectory, with the GETomics approach underscoring how lifetime exposures such as smoking can induce epigenetic changes, thereby exacerbating or triggering COPD [184]. These interactions between genetic predispositions and epigenetic modifications such as smoking-related DNA methylation, can intensify disease progression [185]. Recognizing these multifactorial influences is essential for disentangling the interplay among aging, epigenetics, and the temporal sequence of metabolic dysregulation in COPD, ultimately paving the way for more targeted, personalized therapeutic interventions [183,184,185].

### 6.6. Metabolomics and Lung Inflammation in Metabolic Dysregulation

Metabolomics, the study of small molecules in biological samples, offers insights into the metabolic dysregulation associated with COPD, particularly in relation to lung inflammation and metabolic pathways. COPD is associated with significant alterations in multiple metabolic pathways, including amino acid metabolism, energy production, and lipid metabolism. These disruptions contribute to systemic inflammation and oxidative stress, both central features of COPD pathogenesis [186,187]. Specifically, the dysregulation of amino acids such as histidine, creatine, and threonine has been documented, with these metabolites showing an inverse correlation with inflammatory markers like IL-6. Moreover, COPD patients exhibit an imbalance between aerobic and anaerobic energy metabolism, often favoring anaerobic pathways, a shift that accelerates lung injury and inflammation and is linked to disease progression [188]. This altered energy metabolism highlights potential therapeutic targets to manage COPD more effectively. Lipid metabolism is likewise notably affected, as changes in lipid mediators and synthesis pathways not only contribute to inflammation but also fulfill the heightened energy requirements of lung tissue, driving anabolic processes that further exacerbate disease. Additionally, sphingolipid dysregulation and alterations in other lipid molecules have been tied to specific COPD phenotypes, such as reduced lung function and emphysema [189].

Oxidative stress plays a crucial role in COPD, with metabolomic studies highlighting sex-associated differences in oxidative stress markers. Females with COPD show enhanced metabolic dysregulation related to oxidative stress compared to males, suggesting potential sex-specific therapeutic approaches. The autotaxin–lysoPA axis is also implicated in oxidative stress and inflammation in COPD [190].

Metabolomic analyses have identified significant alterations in the arginine and trans-sulfuration pathways, which are involved in nitric oxide production and oxidative stress. These changes are associated with increased levels of inflammatory markers and may serve as potential biomarkers for COPD management [191]. Metabolomic profiling offers potential for developing diagnostic tools to differentiate COPD from other respiratory conditions, such as asthma. The unique metabolic signatures identified in COPD patients could lead to more accurate diagnoses and personalized treatment strategies [192].

## 7. Clinical Implications of the Metabolic–Inflammatory Axis

### 7.1. COPD and Metabolic Syndrome

Metabolic syndrome (MetS) is a multifaceted condition that is clinically characterized by various risk factors, including abdominal obesity (BMI > 30 kg/m^2^), elevated blood pressure, a dyslipidemic profile conducive to atherogenesis, and impaired fasting blood glucose, with or without insulin resistance [193]. The International Diabetes Federation (IDF) has established a general consensus indicating that neither cigarette smoke nor COPD are recognized as canonical risk factors for metabolic syndrome, nor is there definitive mechanistic evidence supporting a causal relationship between these conditions [121]. However, recent clinical findings suggest a strong association [194].

More than 30% of individuals diagnosed with COPD present with one or more components of MetS, a fact that might significantly impair patient prognosis [195]. COPD patients with MetS are more likely to be female, have a higher body mass index, and demonstrate higher FEV1 scores compared to those without MetS [123]. The heightened prevalence of MetS among patients with less severe airflow obstruction is likely attributable to the weight loss commonly observed in advanced stages of COPD, in conjunction with increased cardiovascular-related mortality associated with MetS in this population [196]. As a result, these patients may experience earlier death due to cardiovascular disease, preventing them from reaching end-stage COPD [195,197]. Moreover, insulin resistance is more prevalent in COPD patients with metabolic syndrome, which facilitates the progression to T2DM [117].

The etiology of MetS associated with COPD is complex, with several key factors contributing to its development, including inflammatory mediators, oxidative stress, and sedentary lifestyle [122]. There is compelling evidence indicating that the pathophysiology of COPD and its associated comorbidities, such as MetS, is profoundly influenced by elevated oxidative stress and the systemic dissemination of lung inflammation [121]. This dissemination, often referred to as “spill-over”, occurs primarily due to increased membrane permeability; however, direct injury within the pulmonary environment can also arise from oxidants present in cigarette smoke, alongside heightened levels of reactive oxygen species and reactive nitrogen species generated by both pulmonary and systemic inflammatory responses [144]. In contrast, oxidative stress related to MetS predominantly results from the activation of specific biochemical pathways, including mitochondrial oxidative metabolism, elevated cellular synthesis due to inflammation, depletion of antioxidant systems, and lipid peroxidation, which is frequently observed in individuals with obesity [198]. Owing to its pro-inflammatory properties, oxidative stress has been proposed as the most likely cause of the elevated risk of cardiovascular comorbidity in both MetS and COPD [199].

As previously noted, proinflammatory cytokines, such as IL-1β, IL-6, TNF-α, and CRP, are persistently elevated in both serum and airway levels due to lung inflammation associated with COPD, and they also play a role in the development of T2DM [121]. Additionally, oxidative stress resulting from cigarette smoke or the body’s inflammatory response further exacerbates insulin resistance [200]. It has been suggested that a vicious cycle exists between MetS and COPD: as lung function declines in COPD patients, increased physical inactivity heightens the risk of weight gain, which, in turn, accelerates lung function deterioration and further limits physical activity [95]. In contrast to patients with COPD who are not obese, these patients exhibit less tolerance to exercise and more severe dyspnea [201].

The inflammation of adipose tissue plays a pivotal role in the relationship between COPD and MetS. In macrophages, leptin functions as an acute-phase reactant, enhancing the secretion of proinflammatory cytokines such as TNF-α, IL-6, and IL-12 [202]. The inflammatory state is thereby encouraged by a feedback loop that raises leptin expression in adipose tissue and circulating leptin [98,203]. This association elucidates the intricate relationship between obesity and impaired cytokine production, increased levels of acute-phase reactants, and persistent proinflammatory signaling pathways, and it underscores the increased susceptibility to inflammatory diseases and immunity-related conditions [204]. Moreover, studies found that smokers had lower levels of leptin than non-smokers [205], while another study hypothesized that nicotine might directly affect insulin resistance by raising the levels of leptin in the blood [206]. All of these results suggest that the main factors influencing the onset and clinical progression of COPD and MetS are low-grade systemic inflammation and hormones linked to adipose tissue.

### 7.2. COPD and Type 2 Diabetes Mellitus

**Figure 4:**
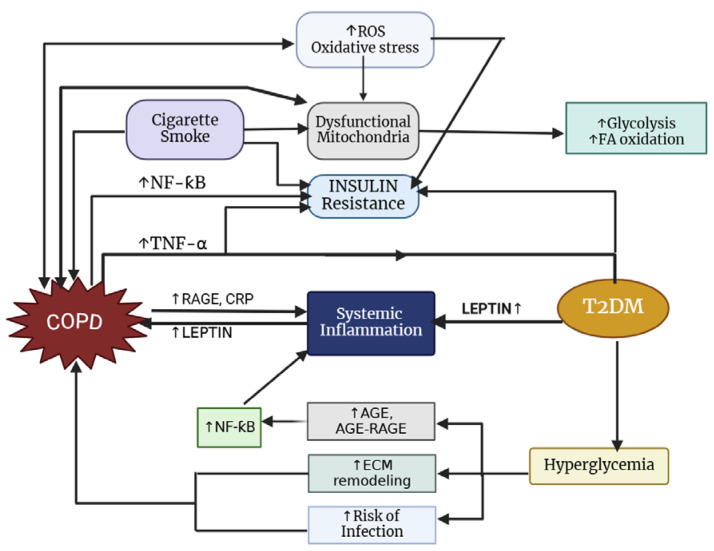
Pathways connecting COPD to type 2 diabetes mellitus.

The prevalence of T2DM among individuals with COPD varies by study; however, it is generally higher, with approximately 18.7% of COPD patients affected compared to 5–10% in the general population [207,208]. Notably, there is a reciprocal relationship between these two conditions (Figure 4), as diabetes commonly coexists with COPD and patients with COPD face an increased risk of developing diabetes [209]. According to Oh et al. [210], smoking significantly increases the risk of diabetes, likely due to the worsening of insulin resistance caused by systemic inflammation and/or oxidative stress associated with smoking. Additionally, overweight and obese individuals face a greater likelihood of developing diabetes [211].

Impaired lung function is one of the most common comorbidities among individuals diagnosed with T2DM [209]. According to Kinney et al. [212], there is a direct link between the severity of diabetes and the drop in FEV1 and FVC. There are several connections between diabetes and deterioration of lung function, including insulin resistance, glucotoxicity, and systemic inflammation. Inflammatory mediators like TNF-α, IL-6, and CRP are persistently elevated in T2DM [193], which may increase lung vascular permeability [213]. It has also been demonstrated that elevated glucose levels in airway secretions can lead to lung function impairment [214]. Hyperglycemia can also lead to the production of pro-inflammatory glycosylation endproducts (AGEs), which can hasten lung complications [215].

By promoting collagen release, proliferation, and contractions of airway smooth muscle cells through β catenin signaling, insulin remodels the pulmonary compartment and contributes to airway hyperresponsiveness [216]. Alveolar hypoxia and the ensuing hypoxemia (systemic hypoxia) are more likely to occur in the setting of COPD when pulmonary function deteriorates over time [139]. Diabetic patients diagnosed with COPD frequently experience hyperglycemia, occurring approximately 80% of the time during their hospital stay, a fact that is correlated with extended hospital stays and an increased risk of mortality when compared to non-diabetic patients [217,218].

In vivo research has shown that chronic hyperglycemia can cause endothelial dysfunction in diabetic patients’ blood vessels through the excessive production of ROS, in addition to systemic inflammation [219]. Elevated levels of reactive oxygen species induced by hyperglycemia can trigger cellular stress, including pathways mediated by NFκB and MAPK, which may adversely affect pulmonary function [220]. Furthermore, hyperglycemia can lead to the presence of glucose in airway secretions, potentially increasing the risk of pulmonary infections by making the respiratory tracts more susceptible to infectious exacerbations [214,221]. Lastly, hyperglycemia-induced oxidative stress and inflammation can cause sarcomeric damage by activating proteolytic machinery, which results in contractile protein wasting and, ultimately, a reduction in the diaphragm fibers’ ability to generate force in patients with COPD [222].

T2DM and insulin resistance are also connected to elevated leptin levels. The percentage of obesity, hypertension, and endothelial dysfunction is higher in this particular population, and hyperleptinemia is linked to an increased risk of cardiovascular diseases (CVD) [223]. As previously stated, leptin’s impact on glucose homeostasis in obese or insulin-resistant individuals is linked to POMC-expressing neurons in the hypothalamic arcuate nucleus (ARC). Studies on animals with type 2 diabetes indicated that leptin replacement therapy improved insulin resistance and suppressed hepatic gluconeogenesis and fasting hyperglycemia [224]. T2DM, macroangiopathy, and insulin resistance have all been generally associated with hyperleptinemia. It is noteworthy that certain antidiabetic medications (metformin, pioglitazone, sitagliptin, liraglutide, and empagliflozin) have demonstrated the capacity to lower leptin levels [225,226], yet the comprehensive clinical implications of these medications remain to be fully elucidated. Moreover, existing research highlights that the distribution of adipose tissue plays a significant role in the development of insulin resistance, independent of the overall level of obesity [227].

### 7.3. COPD and Cardiovascular Disease

Numerous studies have shown that COPD patients have a higher incidence and prevalence of CVD than general population, making them especially susceptible to cardiovascular morbidity and mortality [228]. Research indicates that among patients with COPD, mortality from CVD is more prevalent than that from respiratory failure [229]. Moreover, patients with both COPD and CVD experience increased dyspnea and decreased exercise tolerance, along with a higher likelihood of hospitalization for either condition [229].

Common risk factors for both diseases include aging, cigarette smoking, and physical inactivity [230]. Hypoxemia, oxidative stress, lung hyperinflation, pulmonary hypertension, hypoxemia, and systemic inflammation represent key pathophysiological connections between cardiovascular disease and COPD [231]. An experimental study involving healthy participants found a significant reduction in left ventricular stroke volume, along with an increase in the end-diastolic radius curvature of the interventricular septum, linked to elevated dynamic hyperinflation induced by expiratory pressure [232]. The primary contributors to hypoxemia, pulmonary hypertension, and the subsequent development of right and left heart failure are ventilation/perfusion mismatches caused by progressive airflow obstruction and emphysema [231].

Low-grade inflammation appears to contribute significantly to the pathogenesis of cardiovascular comorbidities via the enhancement of atherosclerotic plaque formation and rupture, the proliferation of smooth muscle cells that results in increased arterial stiffness, increased platelet aggregation, the exacerbation of endothelial dysfunction, and a reduction in progenitor endothelial cells (CD^34+^) [233]. In patients with stable COPD, exogenous stimuli have been shown to trigger both innate and adaptive activation [234], but cardiovascular events are especially risky during acute exacerbations. A five-day temporary elevated risk for acute myocardial infarction was linked to an acute exacerbation of COPD [235]. Procoagulant activity is also elevated in COPD patients due to inflammation. Coagulation, in turn, intensifies inflammation, and both are closely linked to the pathophysiology of atherothrombosis [236].

Cigarette smoke exposure is a major risk factor for the onset and progression of atherosclerosis due to its proinflammatory effects and ability to induce oxidative stress [237]. Studies have shown that smoking enhances systemic inflammation, leading to the destabilization of vulnerable plaques and promoting a prothrombotic environment in the vasculature [238,239]. Crucially, smoking causes both dyslipidemia and systemic inflammation. This interaction exacerbates dyslipidemia, resulting in detrimental cycles, while dyslipidemia enhances the availability of oxidized low-density lipoprotein (LDL), thereby contributing to the formation of atherosclerotic plaques, persistent systemic inflammation, and pulmonary dysfunction [237,239].

Additionally, leptin controls vascular and cardiac function through a NO-dependent mechanism [240]. Additionally, the onset of insulin resistance and hypertension has been associated with leptin resistance [241]. Hyperleptinemia is a strong predictor of acute myocardial infarction (AMI) and is considered an independent risk factor for coronary artery disease (CAD) [242]. In patients with CAD, elevated circulating leptin levels have been linked to a range of complications, including impaired diastolic function, short-term cardiac remodeling, acute coronary syndrome, stroke, heart failure, and cardiac death [243,244,245,246,247].

Emerging evidence suggests that long-acting muscarinic antagonists (LAMAs) and long-acting beta-agonists (LABAs) may potentially increase the risk of developing cardiovascular disease in susceptible populations or worsen preexisting cardiovascular disease, even though they have long been the standard pharmacological treatment for COPD [247,248]. While LABAs have a tendency to be beta 2-selective, the primary concern is with beta-adrenergic therapy, which can increase sympathetic nervous system activation [249] and cause cardiac rhythm abnormalities. LABAs and inhaled corticosteroids (ICSs) are frequently used in patients who are more likely to experience exacerbations. ICSs may exacerbate heart failure, ventricular arrhythmias, and atrial fibrillation, even though they are believed to lower cardiovascular mortality in COPD patients [248]. Clinical trial data indicate that inhaled COPD treatments do not significantly increase the risk of CVD, at least for those without cardiovascular comorbidities [248]. According to Gershon et al. [250], new users of LABA and LAMA had higher cardiovascular risk, as indicated by hospitalizations or ER visits for CVD. As a result, individuals with COPD and a history of heart disease may be more susceptible to atrial fibrillation and other cardiac conditions.

### 7.4. COPD, Muscle Wasting, and Dysfunction

Skeletal muscle wasting and dysfunction is another prevalent “scenario” associated with COPD, significantly impacting patients’ survival and life quality [251]. The loss of muscle mass impacts approximately 40% of COPD patients, with both severity and prevalence of muscle wasting being more pronounced in those who are in advanced stages of the disease [97,119,252]. Research has established that, independent of the decline in pulmonary function, a low FFMI and diminished quadricep strength serve as critical predictors of mortality in COPD patients [253,254], emphasizing the role that muscle mass and functionality play within the broader pathophysiological context. Moreover, patients with COPD experience phenotypic alterations in their muscle fibers, characterized by a reduction in the proportion of slow, oxidative type I fibers and an increase in fast glycolytic type II fibers [255,256,257,258].

Insulin plays a vital role in the regulation of muscle protein metabolism, and when insulin resistance occurs, it can disrupt the delicate balance between protein synthesis and breakdown [120]. As previously highlighted, individuals diagnosed with COPD exhibit increased levels of insulin resistance in comparison to non-smoking matched controls [114]. Additionally, research indicates a significant negative correlation between insulin resistance and quadricep strength in both young [259] and old [260] adults who do not have COPD or diabetes mellitus. Although the mechanisms governing this association are complex, increased baseline insulin resistance is associated with a faster decline in skeletal muscle strength over the ensuing three years [261]. Moreover, insulin is essential for sustaining mitochondrial functional activity because it promotes the synthesis of proteins in the mitochondria [262].

Insulin resistance results in impaired vasodilation and endothelial dysfunction, which can impede the increase in blood flow to skeletal muscles during moderate exercise, ultimately contributing to exercise limitations [120]. Insulin resistance is linked to hyperglycemia, which affects skeletal muscle on its own. Hyperglycemia, when examined in vitro, activates pathways related to skeletal muscle atrophy through mechanisms such as caspase 3 activation, degradation of myofibrillar proteins, and the ubiquitin–proteasomal degradation pathway [263,264]. It is plausible that shared etiological factors account for the observed correlation between peripheral muscle weakness and insulin resistance [120]. Cigarette smoking decreases glucose absorption and hinders the action of insulin [265]. Additionally, physical inactivity, even in individuals with mild airflow obstruction, has been associated with quadricep wasting in COPD patients and is recognized as a significant risk factor for IR [197]. Intermuscular adipose infiltration is linked to decreased muscle strength and compromised physical function and is frequently seen in individuals with insulin resistance [266] or COPD [267].

It is important to note that both COPD and T2DM exhibit similar metabolic and mitochondrial alterations in skeletal muscle. These changes include shifts in fiber type, a reduction in oxidative capacity, decreased mitochondrial density, and alterations in peroxisome proliferator-activated receptor gamma co-activator 1 [120]. Moreover, both conditions show excessive production of ROS and increased oxidative damage to mitochondrial DNA [120].

The hypothesis suggesting that cachexia and muscle wasting in COPD caused by altered energy balance may arise from dysfunctions in the leptin feedback mechanism has been explored extensively in the literature [268]. Nonetheless, no statistically significant correlation has been identified between circulating leptin levels and the activated TNF-α system [269]. Researchers have not been able to demonstrate a consistent elevation of leptin levels in cachexic stable COPD patients [118]. However, when accounting for factors such as fat mass and the use of oral corticosteroids, certain studies have reported a significant partial correlation coefficient between leptin and soluble tumor necrosis factor receptor 55 (sTNF-R55) [269]. Moreover, leptin levels were determined to be linked to fat mass in patients with emphysematous COPD, which appears to support the feedback mechanism known to regulate body weight, while this correlation was not observed in patients with chronic bronchitis [25]. While it appears to be physiologically regulated, low levels of the hormone may be responsible for an increased risk of pulmonary infections in COPD patients [118].

Takabatake et al. [270] examined the circadian rhythm of circulating leptin in individuals with COPD to gain a deeper understanding of its dynamics. The study revealed that this circadian pattern is preserved in COPD patients with normal BMI, whereas it is absent in those exhibiting cachexia [269]. These findings indicate that the disruption of the physiological leptin release pattern in cachectic COPD patients may hold clinical significance concerning pathophysiological features, including abnormalities in the hypothalamic–pituitary axis and the autonomic nervous system, and this loss of rhythm may serve as a compensatory mechanism to help maintain body fat content [270].

### 7.5. COPD and Obesity

The “obesity paradox” in COPD refers to the intriguing observation that overweight and moderately obese COPD patients exhibit better survival outcomes compared to normal-weight individuals, particularly those with severe bronchial obstruction. This paradoxical relationship appears to diminish at extremely high BMI levels (>40 kg/m^2^), suggesting a U-shaped association between BMI and mortality [271,272]. Potential mechanisms behind this paradox include beneficial mechanical changes in chest-wall dynamics that reduce lung hyperinflation. However, confounding factors such as reverse causation and variability in emphysema severity complicate interpretations [271].

Although leptin, a key regulator of body weight and energy balance, is generally lower in COPD patients compared to healthy controls, it correlates primarily with BMI and body composition rather than inflammation or cachexia-related markers. Notably, leptin levels do not consistently correlate with COPD-associated cachexia, questioning its role as a direct therapeutic target for managing weight loss in these patients. Due to these complexities, further research is essential to delineate specific obesity phenotypes, clarify leptin’s mechanistic role, and evaluate the potential of leptin sensitizers or other targeted interventions to address obesity-related complications in COPD management [273].

Leptin plays a crucial role in the pathophysiology of COPD, notably influencing systemic inflammation, body composition, and disease progression. Elevated leptin levels observed in COPD patients, particularly during exacerbations, correlate closely with inflammatory markers such as CRP and TNF-α, indicating its involvement in systemic inflammatory responses [274]. In stable conditions, leptin concentrations correlate with body mass index and percent body fat, demonstrating preserved leptin regulation despite weight changes; however, this correlation weakens during disease exacerbations, suggesting a disruption in its normal regulatory function [275]. Gender differences are also evident, with higher leptin levels typically observed in women with COPD, particularly among those who are overweight, potentially impacting inflammation and disease progression uniquely [87]. Furthermore, age-related leptin resistance can amplify systemic inflammation, metabolic dysregulation, and muscle wasting in older COPD patients, exacerbating cachexia and reducing quality of life. Given these complex interactions, leptin not only holds promise as a biomarker for inflammation and disease monitoring but also represents a potential therapeutic target. Future research focusing on age- and gender-specific leptin dynamics could further clarify its mechanistic role and optimize personalized treatment strategies in COPD management [276].

## 8. COPD in Low- and Middle-Income Countries

Chronic obstructive pulmonary disease constitutes a considerable public health challenge in low- and middle-income countries (LMICs), where more than 90% of deaths attributable to COPD occur. Unlike in high-income nations, where tobacco smoking is the predominant risk factor, COPD in LMICs is strongly associated with indoor air pollution, occupational exposures, and inadequate healthcare infrastructure [277]. Many households in these regions rely on biomass fuels (charcoal, wood, and agricultural waste) for cooking and heating purposes, leading to chronic exposure to fine particulate matter and toxic pollutants. Prolonged inhalation of these pollutants triggers persistent airway inflammation, oxidative stress, and accelerated lung function decline, exacerbating COPD progression [278]. Biomass smoke exposure disproportionately impacts women and children due to their domestic roles, causing unique inflammatory patterns distinct from cigarette-induced COPD. Specifically, biomass smoke induces inflammation primarily through the IL-17F/IL-17RC pathway, resulting in bronchial hyperresponsiveness, bronchial anthracofibrosis, thicker pulmonary arterial intima, significant fibrosis, and typically less emphysema compared to tobacco-induced COPD [279]. Biomass-induced COPD is also characterized by lymphocyte-dominant airway inflammation and increased pigment deposition, reflecting different immune responses compared to the neutrophil-driven inflammation seen in cigarette-smoke COPD [279]. Additionally, pro-inflammatory diets, indicated by high Dietary Inflammatory Index (DII) scores, exacerbate COPD risk and correlate with decreased lung function. Addressing COPD in these regions necessitates interventions focused on improved ventilation to mitigate indoor biomass smoke exposure and dietary modifications emphasizing anti-inflammatory and antioxidant-rich foods, strategies that could significantly reduce disease severity and improve public health outcomes [280].

Additionally, rapid urbanization has led to increased exposure to industrial pollutants and vehicle emissions, further contributing to chronic respiratory inflammation [281].

Inflammatory markers such as CRP, IL-6, and TNF-α are consistently elevated in COPD patients from LMICs, indicating a higher systemic inflammatory burden. Frequent respiratory infections, including tuberculosis and bacterial pneumonias, are more common in these regions and further exacerbate airway inflammation [282]. Malnutrition, another prevalent issue in LMICs, weakens immune function and contributes to a heightened inflammatory state, worsening COPD symptoms and increasing the risk of severe exacerbations. Additionally, diets characterized by lower energy and protein intake, as well as deficiencies in micronutrients such as calcium, potassium, iron, folate, zinc, vitamin B6, retinol, and niacin, exacerbate the risk of malnutrition and negatively impact disease outcomes [283,284]. Poor dietary quality in COPD patients is frequently linked to disease-related symptoms such as breathlessness, anorexia, depression, and social factors including poverty and isolation. Western-style diets, high in processed meats and low in fiber-rich foods, further contribute to COPD risk and accelerated pulmonary decline; conversely, diets rich in fruits, vegetables, antioxidants, and dietary fiber have protective effects, particularly noted in middle-aged and elderly populations [283]. In LMICs, adherence to healthy dietary guidelines remains low due to economic, social, and cultural barriers that enhance the availability of unhealthy foods [285]. Suboptimal dietary practices significantly contribute to the global burden of non-communicable diseases (NCDs), with elevated sodium intake, low whole grain consumption, and inadequate fruit intake identified as leading risk factors [283]. Given these challenges, there is an urgent need for targeted nutritional interventions and policy frameworks in LMICs that prioritize healthy food access, improve dietary adherence, and address socio-economic determinants to effectively mitigate COPD and associated NCDs [278].

Additionally, metabolic comorbidities such as diabetes and cardiovascular disease, which are often underdiagnosed or poorly managed in LMICs, further contribute to disease progression.

A major challenge in managing COPD in LMICs is underdiagnosis and limited access to effective treatment. Spirometry, the gold standard for COPD diagnosis, is often unavailable in primary healthcare settings, leading to misclassification of COPD as asthma or chronic bronchitis [286]. Additionally, bronchodilators, inhaled corticosteroids, and pulmonary rehabilitation programs remain unaffordable for many patients due to financial constraints and limited healthcare funding. As a result, COPD patients in LMICs experience higher rates of severe exacerbations and hospitalizations, increasing the overall mortality burden [287].

To reduce the impact of COPD in LMICs, urgent public health interventions are needed [277]. Reducing household and outdoor air pollution through clean cooking technologies, stricter industrial emissions policies, and improved air quality monitoring could significantly lower the inflammatory burden in COPD patients. Expanding access to early diagnosis through training healthcare providers and increasing the availability of cost-effective medications are also crucial steps [1,6]. Additionally, public health initiatives promoting tobacco control, nutritional support, and vaccination programs (influenza and pneumococcus) could help mitigate COPD-related complications. Addressing these challenges holistically will be key to reducing COPD morbidity and mortality in low-income countries [1,8].

## 9. Therapeutic Management and Future Directions

COPD management currently revolves around symptom relief, exacerbation reduction, and improvement of overall quality of life. Conventional therapeutic strategies primarily include pharmacological treatments such as LAMAs, LABAs, inhaled corticosteroids, and systemic therapies for advanced disease stages. Pulmonary rehabilitation programs and lifestyle modifications, particularly smoking cessation and exercise, remain foundational. Despite the prevalence of COPD, current treatments like glucocorticoids and bronchodilators have limitations, including side effects and limited efficacy in halting disease progression. Consequently, there is significant interest in developing novel therapeutic agents that target the underlying mechanisms of COPD more effectively.

Recent research has focused on molecular targeted therapies that address specific pathways involved in COPD. Thioredoxin (Trx) is a promising agent that regulates redox status and protease/anti-protease balance, blocks NF-κB and MAPK signaling pathways, and improves steroid insensitivity by inhibiting macrophage migration inhibitory factor (MIF) production [288,289,290]. These mechanisms differ from traditional glucocorticoid-based treatments, offering a novel approach to managing COPD. Given the limited response of COPD to corticosteroids, novel anti-inflammatory drugs are being explored. These include agents that inhibit the recruitment and activation of inflammatory cells such as macrophages, neutrophils, and T-lymphocytes. Some promising candidates include chemokine receptor antagonists, matrix metalloproteinase inhibitors, and p38 MAPK inhibitors [288,289,290]. The monoclonal antibody dupilumab, which targets inflammatory pathways, has shown success in clinical trials, highlighting the potential of biologic agents in COPD treatment [291].

Antioxidants and anti-fibrotic compounds are also under investigation for their potential to mitigate oxidative stress and tissue remodeling in COPD. Compounds like N-acetyl-L-cysteine and other antioxidant enzyme mimetics are being studied for their ability to reduce oxidative damage. Additionally, anti-fibrotic agents aim to prevent airway remodeling, a key feature of COPD progression [292].

Glucagon-like peptide-1 receptor agonists (GLP-1RAs) and leptin sensitizers have shown potential, particularly for COPD patients with comorbid T2DM [293]. GLP-1RAs reduce inflammatory responses, oxidative stress, airway remodeling, and protease/anti-protease imbalance, improving airway mucus homeostasis. Studies indicate that GLP-1RAs significantly lower the risk of moderate and severe COPD exacerbations compared to other diabetes medications like dipeptidyl-peptidase 4 inhibitors (DPP-4i) and sulfonylureas, resulting in improved clinical outcomes and reduced healthcare utilization [294,295]. Furthermore, GLP-1 analogs are associated with improved pulmonary outcomes, including a lower risk of pneumonia, reduced oxygen dependence, and decreased all-cause mortality in COPD patients receiving single-inhaler triple therapy (SITT) [296]. GLP-1RAs also reduce leptin levels, potentially contributing to anti-inflammatory effects and mitigating inflammation-related lung damage, suggesting benefits beyond their glucose-lowering capabilities. While direct evidence on leptin sensitizers specifically in COPD is limited, the modulation of leptin levels by GLP-1RAs suggests a potential pathway for reducing inflammation and improving immune function in COPD patients [297]. This could be particularly relevant in managing COPD exacerbations and improving overall lung function.

Further studies could help refine treatment guidelines and improve patient outcomes by leveraging the dual benefits of these agents on metabolic and respiratory health [293].

Recent advancements have demonstrated the potential of interventional therapeutic approaches in managing COPD, particularly in advanced stages where pharmacological treatments alone may not suffice. Interventional therapies, including bronchoscopic lung volume reduction (BLVR), endobronchial valves, and lung volume reduction surgery (LVRS), have increasingly gained attention for their ability to substantially improve patient outcomes.

BLVR using endobronchial valves has shown significant improvements in lung function, exercise tolerance, and quality of life in patients with severe emphysema. Studies report increased forced expiratory volume in 1 s (FEV1), improved six-minute walking distance, and reduced dyspnea scores post-procedure [298]. The procedure is generally safe, with no significant increase in pneumonia rates or mortality compared to control groups, although there is a risk of complications such as pneumothorax [299].

LVRS has been shown to improve survival, lung function, and quality of life in selected patients with upper lobe-predominant emphysema and low exercise capacity. However, it is associated with significant complications, prolonged hospital stays, and high costs [300,301]. A randomized controlled trial comparing LVRS and BLVR found no substantial difference in outcomes at one year, suggesting that BLVR may be a viable alternative for patients who are suitable for both procedures [300].

Improving the quality of life (QoL) for patients with COPD involves a diverse array of therapeutic interventions. Self-management interventions (SMIs), particularly those targeting symptom control, physical activity, and mental health, have consistently demonstrated efficacy in enhancing health-related quality of life (HRQoL) and reducing emergency department visits across various patient subgroups, including those with severe symptoms and high body mass indices [302,303]. Telehealth approaches, while promising, have yielded mixed results, with only limited studies reporting significant improvements compared to standard care [304]. Although palliative care has not shown substantial direct benefits in QoL, it effectively reduces acute healthcare utilization, such as hospitalizations and intensive care admissions. Psychological interventions, especially cognitive behavioral therapy, have exhibited effectiveness primarily in reducing anxiety symptoms, though their overall impact on QoL remains limited. Integrated disease management programs, characterized by their multidisciplinary and multi-component nature, have emerged as effective strategies for improving disease-specific QoL, enhancing exercise capacity, and decreasing hospital admissions [305]. Additionally, web-based supportive interventions have proven valuable as supplementary options, significantly improving QoL when combined with usual care, despite not surpassing the effectiveness of face-to-face treatments [302].

Looking forward, therapeutic management strategies are likely to become increasingly personalized, with a greater emphasis on identifying phenotypes that respond best to specific interventional treatments. Technological advancements, alongside more refined patient-selection criteria, will continue to enhance the precision and effectiveness of COPD management, ultimately leading to improved patient-centric outcomes and reduced disease burden.

## 10. Conclusions

COPD extends well beyond the pulmonary compartment and is profoundly influenced by the interplay of metabolic, inflammatory, and epigenetic pathways, all of which can be further modulated by aging. Leptin and insulin, both key regulators of energy homeostasis, become dysregulated in COPD, contributing to muscle wasting, insulin resistance, and an increased risk of cardiometabolic comorbidities. Epigenetic modifications such as DNA methylation can amplify or alter these processes, shaping disease trajectory and therapeutic responses, especially when combined with environmental exposures like smoking or poor nutritional status.

The existing evidence underscores a pressing need for further research into how these metabolic and epigenetic factors converge to influence different COPD phenotypes, potentially uncovering novel biomarkers and patient-specific treatment strategies. By incorporating multi-omics approaches and focusing on the metabolic–inflammatory–epigenetic axis, personalized interventions and earlier therapeutic targets may emerge. Ultimately, refining our understanding of these multifaceted processes offers the prospect of reducing disease exacerbations and improving both respiratory and metabolic health outcomes for individuals suffering from this debilitating condition.

## Figures and Tables

**Table 1:** Key studies on leptin and insulin dysregulation in COPD patients.

Author (Year)	Main Outcomes
Bolton et al. (2007) [93]	COPD patients had greater insulin resistance than the healthy subjects
Breyer et al. (2011) [87]	Leptin is increased in COPD women when compared to healthy women and compared to COPD men and to a greater extent in overweight women with COPD
Breyer et al. (2012) [88]	Female COPD subjects had higher leptin and higher ratios of leptin/FM and leptin/adiponectin compared with male COPD subjects
Eker et al. (2010) [114]	Leptin levels were lower in COPD patients compared to controls. COPD patients had higher HOMA-IR scores
Minas et al. (2011) [117]	Patients with COPD and MetS show higher leptin levels and greater insulin resistance compared to those without MetS.
Takabatake et al. (1999) [118]	Serum leptin was lower in COPD patients compared to healthy controls, yet it remains physiologically regulated even amidst cachexia.
Schols et al. (1999) [25]	Emphysematous patients had lower leptin concentrations compared with bronchitic patients.
Broekhuizen et al. (2005) [119]	Leptin is detectable in COPD sputum, inversely correlates with plasma leptin, and is significantly linked to CRP and TNF-α.
Vernooy et al. (2009) [85]	Ex-smokers, with or without severe COPD, show higher leptin expression in bronchial epithelial cells and alveolar macrophages than never-smokers.
Gaki et al. (2011) [90]	In patients with COPD, both BODE index and FFMI presented significant positive and negative associations, respectively, with leptin levels.
Wells et al. (2016) [120]	Insulin resistance inversely correlates with quadricep strength, and patients with quadricep weakness exhibit significantly higher insulin resistance

BODE: body mass index, airflow obstruction, dyspnea, and exercise capacity; HOMA-IR: homeostasis model assessment insulin resistance; MetS: metabolic syndrome.

## Data Availability

The data presented in this study are available from the corresponding author upon request.
